# Clinical observation of modified gastric tube in middle and lower thoracic esophageal carcinoma surgery

**DOI:** 10.1186/s13019-019-0967-y

**Published:** 2019-07-30

**Authors:** Bo Liu, Wei Wang, Tao Liang

**Affiliations:** Department of Thoracic Surgery, The First Affiliated Hospital of Anhui University of Chinese Medicine, No.117 Meishan Road, Hefei, 230031 Anhui People’s Republic of China

**Keywords:** Middle and lower thoracic esophageal cancer, Modified gastric tube, Pulmonary function

## Abstract

**Background:**

A clinical case-control study was conducted to analyze the short-term efficacy of modified gastric tube in surgery for middle (mid)- and lower- thoracic esophageal carcinoma compared with the conventional gastric tube and its effect on postoperative pulmonary function.

**Methods:**

A total of 70 patients with mid- and lower-thoracic esophageal cancer who underwent esophagectomy between October 2012 and September 2018 in our hospital were recruited in the study. They were randomly divided into a modified gastric tube group (*n* = 35) and a conventional gastric tube group (*n* = 35). The operation time, intraoperative blood loss, number of intraoperative lymph node dissection, gastrointestinal decompression time and postoperative hospital stay were recorded. The operation results and complications were recorded, and the pulmonary function was recorded at 3 days before surgery and 6 weeks after surgery.

**Results:**

The operation time in the modified gastric tube group was significantly lower than that in the gastric tube group (*P* < 0.05). There were no anastomotic leakage or death occurred in the modified gastric tube group. There was 1 case of anastomotic leakage in the conventional gastric tube group. The pulmonary function in both groups was improved at 6 weeks after surgery, but there was no significant difference between both groups (*P* > 0.05).

**Conclusion:**

Modified gastric tube has a good clinical application value compared with gastric tube for patients with mid- and lower-thoracic esophageal cancer. It is easy and safe, and can shorten the operation time without aggravation of pulmonary function after surgery.

## Background

The incidence of esophageal adenocarcinoma has increased over the last 30 years. The mid- and lower- thoracic segments of the esophagus are the most common sites of esophageal cancer, especially in esophageal squamous cell carcinoma [1]. Surgery has traditionally been the preferred treatment for early and middle stage esophageal cancer. In esophagectomy, the stomach is the most commonly used conduit for esophageal reconstruction. However, postoperative reflux esophagitis and thoracogastric syndrome usually occur in patients with total gastric replacement, which results in various pulmonary complications, such as pulmonary infection, atelectasis, pulmonary thromboembolism and acute lung injury, affecting respiratory function and postoperative rehabilitation [2, 3]. In recent years, it has become popular to cut the stomach into tubular stomach to replace the esophagus, which not only relieves the symptoms of acid reflux, but also occupies less space in the chest cavity and significantly reduces the influence on respiratory function. In this study, we conducted a clinical case-control study to analyze the short-term efficacy of modified gastric tube in surgery for mid- and lower thoracic esophageal carcinoma compared with the conventional gastric tube and its effect on postoperative pulmonary function.

## Methods

### Study design and patients

This study was approved by the Research Ethics Committee of the First Affiliated Hospital of Anhui University of Chinese Medicine. Written informed consent was obtained from each participant. A total of 70 patients with mid- and lower-thoracic esophageal cancer who underwent esophagectomy between October 2012 and September 2018 at the thoracic surgery department in our hospital were recruited in the study. All patients were diagnosed by upper gastrointestinal angiography and gastroscopy, and without surgery contraindications. They were randomly divided into a modified gastric tube group (*n* = 35) and a conventional gastric tube group (*n* = 35) according to the production methods of tubular stomach. All resections were performed by the same surgical team, patients were treated with the same postoperative regimen during hospitalization. General clinical data of the patients was present at Table 1. There were no significant differences in age, gender, tumor, pathological stage, hypertension, smoking, and drinking between the both groups.

**Table 1:** The comparison with preoperative clinical data (n)

Parameter	Modified gastric tube	Conventional gastric tube	*t/χ2*	*P*
Age	64.06 ± 8.69	65.00 ± 10.12	0.418	0.677
Gender				
Male	31	30	0.128	0.721
Female	4	5		
Tumor				
Middle	26	24	0.280	0.597
Lower	9	11		
Pathological stage				
I	4	3	0.233	0.890
II	11	12		
III	18	20		
Hypertension				
Yes	12	10	0.265	0.607
No	23	25		
Smoking				
Yes	21	23	0.245	0.621
No	14	12		
Drinking				
Yes	13	11	0.254	0.615
No	22	24		

### Surgical methods

Tubular gastric surgery was performed as described previously [4, 5]. For the conventional gastric tube group, surgery was performed under general anesthesia with double-lumen endotracheal tube intubation. Thoracotomy was performed as a standard posterolateral incision on the left. The diseased esophagus and stomach were fully dissociated to the pyloric tube. During this period, it was worth noting that the stomach tissue should not be squeezed, rubbed and the right vascular arch of the gastroomentum should be strictly protected. The esophagus was cut off at 5 cm exceed the tumor edge, the stump of esophageal was disinfected with 0.5% iodide and then inserted into stapling device and closed by means of a purse-string suture. To prepare the gastric tube, the dissociated stomach was flattened, and the anterior 2 to 3 branches of the right vascular arch were preserved. A linear cutter stapler device was used to close the gastric wall of the small curvature of the stomach from 2 to 3 cm above the pylorus to the gastric fundus about 5 cm from the cardia. The seromuscular layer was sutured intermittently with silk suture, and the lesser curvature side of the stomach was completely embedded to make a tubular stomach with 3–4 cm in diameter. A small incision was opened in the anterior wall of the tube stomach, and the stapler was inserted, which combined with the nail holder, completing end-to-side anastomosis between the esophagus and the remnant stomach. Finally, the incision of the anterior wall of the stomach was sutured and the anastomosis was strengthened.

For modified gastric tube group, the esophagus was dissociated, amputated and then placed in stapler holder following the same procedures as the conventional gastric tube group. The lesser curvature of stomach was excised from the fundus toward the pylorus. When the last nail bin distance was left, the small gastric curvature was not completely cut off. A 2 cm incision was made at the partially removed small gastric curvature, and the anastomat was inserted to complete the end-to-side anastomosis between the esophagus and the remnant stomach. Finally, the remaining small curvature of the stomach was removed by a linear cutter stapler device and the anastomosis was strengthened.

### Assessment of related indexes

Operative time, intraoperative blood loss, intraoperative lymph node dissection, gastrointestinal decompression time, and postoperative hospital stay were recorded for all patients. Postoperative anastomotic fistula, gastroesophageal reflux, chylothorax, arrhythmia, pulmonary complications, fat liquefaction of incision and other complications were also recorded. APS-PROSA lung function analyzer was used to detect lung function indicators such as lung vital capacity (VC), maximum ventilation volume (MVV) and forced expiratory volume at the 1st second (FEV1) at 3 days before surgery and 6 weeks after surgery..

### Statistical analysis

All data were analyzed using SPSS 19.0 (SPSS, Inc., Chicago, IL, USA). The quantitative data were expressed as the mean ± standard deviation (SD) and compared using the unpaired Student’s *t-*test. The counting data were expressed by frequency or rate, and the comparison between groups was carried out by Chi-square test. *P* < 0.05 was considered to indicate statistically significant.

## Results

As shown in Table 2, the operation time in the modified gastric tube group was significantly lower than that in the conventional gastric tube group (*P* < 0.05). However, there were no significant differences in intraoperative blood loss, intraoperative lymph node dissection, gastrointestinal decompression time, and postoperative hospital stay between the both groups (*P* > 0.05).

**Table 2:** The comparison of general surgical situation $\left(\overline{\mathrm{x}}\pm \mathrm{s}\right)$

Clinical parameters	Modified gastric tube	Conventional gastric tube	*t/χ*^*2*^	*P*
Operation time (min)	172.43 ± 14.32	182.43 ± 18.49	2.53	0.014
Intraoperative blood loss (ml)	378.29 ± 61.91	371.43 ± 62.22	0.462	0.645
Intraoperative lymph node dissection	13.60 ± 3.32	13.29 ± 3.51	0.385	0.702
Gastrointestinal decompression time (d)	8.43 ± 1.84	8.74 ± 1.88	0.707	0.482
Postoperative hospital stay (d)	22.57 ± 11.26	24.54 ± 18.18	0.545	0.587

The comparison of postoperative complications between both groups was indicated in Table 3. There were no anastomotic leakage or death occurred in the modified gastric tube group. However, there was 1 case of anastomotic leakage in the conventional gastric tube group. Furthermore, no statistically significant difference was observed in anastomotic stenosis, gastroesophageal reflux, emptying disorder, thoracogastric syndrome, chylothorax, arrhythmia, pulmonary complications, and fat liquefaction of incision complications between the both groups.

**Table 3:** The comparison of postoperative complications (n)

Clinical parameters	Modified gastric tube	Conventional gastric tube	*t/χ*^*2*^	*P*
Anastomotic leakage	1	0	1.01	0.314
Anastomotic stenosis	3	4	0.159	0.690
Gastroesophageal reflux	5	7	0.402	0.526
Emptying disorder	4	6	0.467	0.495
Thoracogastric syndrome	7	8	0.085	0.771
Chylothorax	1	1	0	1.0
Arrhythmia	6	5	0.108	0.743
Pulmonary complications	4	5	0.128	0.721
Fat liquefaction of incision	3	2	0.215	0.643

As shown in Table 4, there was no significant difference in the pulmonary function between the both groups at 3 days before surgery (*P* > 0.05). The pulmonary function in both groups was improved at 6 weeks after surgery, but there was no significant difference between both groups (*P* > 0.05).

**Table 4:** The comparison of postoperative pulmonary function $\left(\overline{\mathrm{x}}\pm \mathrm{s}\right)$

	Clinical parameters	Modified gastric tube	Conventional gastric tube	*χ2*	*P*
	VC%	85.10 ± 6.67	86.39 ± 6.88	0.797	0.428
3 days before surgery	MVV%	79.91 ± 6.45	79.53 ± 6.40	0.246	0.807
	FEV_1_%	89.80 ± 10.98	86.03 ± 12.76	4.324	0.190
	VC%	74.50 ± 7.52	73.96 ± 7.80	0.295	0.769
6 weeks after surgery	MVV%	69.31 ± 7.44	68.00 ± 6.99	0.757	0.452
	FEV_1_%	72.77 ± 5.46	68.79 ± 11.25	1.884	0.064

## Discussion

Pulmonary complications occurred in over 30% of patients with esophageal cancer after surgery, which had a serious impact on their respiratory function [6]. Growing data has indicated the pulmonary function of patients with esophageal cancer was significantly impaired after surgery, which affected the postoperative recovery [7, 8]. The traditional method of esophagectomy is to replace the esophagus with the whole stomach, which leads to the occupation of the chest volume when thoracic gastric dilatates. In the negative pressure environment of the thoracic cavity, the dilated stomach can easily affect other organs and compress the expansion of the lung on the operative side, thereby seriously impairing respiratory function of patient [9]. At present, “tubular stomach” has become an option for more and more thoracic surgeons in the treatment for patients with esophageal cancer. The diameter of the tubular stomach is mostly 3–5 cm. This surgical method alleviates thoracogastric syndrome and has little impact on the respiratory function of patients [10]. However, the tubular stomach is often accompanied by a long cut edge and many wounds, and patients are prone to gastric bleeding at the resection margin and residual gastric fistula, which can cause mediastinal infection and even sepsis. In addition, the high operation cost, long operation time and complications have attracted clinicians’ attention, thus there is greater need to improve the operation method [11, 12].

Compared with conventional tubular gastric surgery, modified tubular gastric surgery has significantly shortened operation time [13]. In the modified tubular gastric surgery, the lesser curvature of the stomach to be resected was put into the stapler, anastomosed with the esophageal stump after the highest point of the gastric fundus, and then the remaining small gastric curvature tissue was finally removed. The whole operation process is relatively simple, and only 2–3 cutting and closing operations can complete the tubegastric production, while 4–5 cutting and closing operations are required for the resection of lesions in the conventional tubegastric production. In addition, compared with conventional tubular stomach, modified tubular stomach has fewer incisions, as there is no need to make an additional incision to place an anastomat in the anterior wall of the stomach after the preparation of the modified tubular stomach, which reduces the damage on the gastric blood vessels, maintains the relative integrity of the stomach anterior wall, and ensures to the greatest extent that the blood supply from the right omentum artery of the greater curvature reaches the lesser curvature of the stomach through the submucosal vascular network. Collectively, modified tubular gastric surgery reduces the use of cutting and closing device, the incidence of postoperative complications, and the financial and psychological burden of patients.

Previous studies have shown that tubular gastric surgery has a protective influence on postoperative lung function. The main factors influencing pulmonary function during operation include lung tissue contusion, postoperative pain, injury to diaphragm and chest wall, compression of the lungs by chest and stomach [9, 14]. Our data showed that modified tubular stomach did not increase postoperative lung function damage, shortened the operation time, reduced the influence of anesthetic drugs on respiratory function, and decreased the time of collapse of the lung on the operative side. Besides, the modified tubular stomach will not cause any compression on the lung tissues.

## Conclusions

In summary, modified gastric tube has a good clinical application value compared with gastric tube for patients with mid- and lower-thoracic esophageal cancer. It is easy and safe, and can shorten the operation time without aggravation of pulmonary function after surgery. It is a better esophagus reconstruction method in esophagectomy for mid- and lower-thoracic esophageal cancer in primary hospitals. However, this study has certain limitations, such as a short follow-up period and a small number of cases, so the efficacy and prognosis of the surgery need to be further studied.

## Data Availability

The datasets used and/or analysed during the current study are available from the corresponding author on reasonable request.

## Abbreviations

FEV1: Forced expiratory volume at the 1st second
MVV: Maximum air volume
VC: Patients’ lung capacity
